# Riding the Wave: Reactive Vector-Borne Disease Policy Renders the United States Vulnerable to Outbreaks and Insecticide Resistance

**DOI:** 10.1093/jme/tjab219

**Published:** 2022-01-22

**Authors:** Kyndall C Dye-Braumuller, Jennifer R Gordon, Kaci McCoy, Danielle Johnson, Rhoel Dinglasan, Melissa S Nolan

**Affiliations:** 1 Arnold School of Public Health, University of South Carolina, Columbia, SC, USA; 2 Bug Lessons Consulting, LLC, Denver, CO, USA; 3 CDC Southeastern Center of Excellence in Vector Borne Diseases, Gainesville, FL, USA; 4 University of Florida Emerging Pathogens Institute, Department of Infectious Diseases & Immunology, Gainesville, FL, USA

**Keywords:** vector-borne disease policy, federal funding, insecticide resistance, emerging disease

## Abstract

Funding for vector-borne disease surveillance, management, and research is cyclical and reactive in the United States. The subsequent effects have yielded gross inequities nationally that unintentionally support recurrent outbreaks. This policy forum is comprised of four primary subsections that collectively identify specific areas for improvement and offer innovative solutions to address national inadequacies in vector borne disease policy and infrastructure.

Vector-borne diseases (VBD) are not new threats to public health. Ancient Greek poets and philosophers such as Homer and Aristotle even wrote about the nuisances of biting mosquitoes, flies, and lice—although their role in vectoring pathogens was not known yet (Durden and Mullen 2019). Since this time, millions of people have been at risk for and became infected with viruses, bacteria, and parasites of arthropod origin every year. Despite advances in science, technology, and medicine which have significantly improved the human response and battles against these infectious diseases, we have still not been able to eliminate them. Even in the twenty-first century, the world can seemingly be stopped by a vector-borne disease, as was seen during the Zika pandemic from 2016–2017. In the United States (U.S.), vector-borne disease cases have more than doubled in the past two decades (CDC 2020a). The most important of which, Lyme disease, is responsible for over 300,000 reported cases annually (CDC 2020b, 2021a). Ultimately these numbers are underreported as reported cases typically do not include asymptomatic or misdiagnosed cases.

## Introduction to Policy Making in the U.S.

Policy creation in the U.S. is an ever-evolving process that involves dynamic input and interaction between different groups of people, including elected officials, lobbyists, and stakeholders. In its simplest form, the creation of legislation and its passage into law follows a standardized script. As different national priorities arise, an idea for a new law is generated, and pending initial endorsement, a congressperson from either the House of Representatives or Senate may introduce a bill. Once a bill is introduced, the document moves to a specific committee within the originating congressional house where it is debated and revised. If a consensus can be reached, the document is passed and moves to the opposing congressional house where it follows a similar process of committee review, revisions, and approval. If both the House of Representatives and Senate can pass the same version of a bill, the document then transfers to the President of the United States, whom has the authority to veto a bill or pass it into law. A congressional appeal can override the President’s decision, pending a two-thirds endorsement from both congressional houses. Typically, 5% (2–7%) of bills written and introduced to a committee will make their way successfully through the legislative process and become enacted into law by Congress and the executive branch (GovTrack 2021). Interestingly, congressional productivity is time dependent, with the majority of bills introduced within the first six months of a new, biennially-elected congressional house (GovTrack 2017).

One type of bill that can be introduced is an authorizations act which is defined as, ‘A law that establishes or continues one or more Federal agencies or programs, establishes the terms and conditions under which they operate, authorizes the enactment of appropriations, and specifies how appropriated funds are to be used’ (United States Senate 2021). These authorizations acts can establish or modify new and existing agencies and may recommend the level of funding an agency or program receives. However, once an agency or program has been authorized, a second bill (an appropriations bill) must be introduced and passed to grant the authorized agency or program the actual funds needed to operate. Without funding appropriated, an authorized agency or program cannot function.

Any eligible U.S. resident can influence policy by reaching out directly to their Senators or Representatives. The forms of communication between constituents and congressperson may vary from emails, letters through the mail, phone conversations and in person meetings with congressional staff, or direct one-on-one communication with the legislator. In fact, legislators weigh the topics and concerns of their constituents higher than topics and concerns presented by organizations and special interest groups. Thus, those individuals passionate on a particular topic are encouraged to advocate for their interest directly and frequently.

## Two Decades of Vector-Borne Disease Policy: From West Nile Virus to Zika Virus and Lyme Disease to Powassan Virus

**Fig 1.**
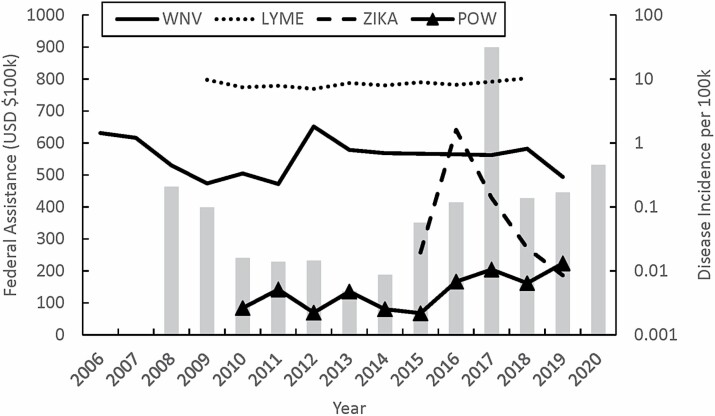
Annual federal funding is reactive to emerging/re-emerging vector-borne disease outbreaks. The website www.usaspending.gov houses a searchable database of federally funded grants issued from 2008 to the present. A search of the terms ‘mosquito’, ‘tick’, and ‘vector-borne’ occurred in February 2021 and resulted in 5,003 grants. The titles of the grants were then manually read, and any grants not related to mosquitoes, ticks, or vector-borne disease were deleted from the database. Results were then sorted by the state of primary activity, and any grants provided to international organizations or primarily funding international research were removed. The final results encompass 798 grants issued between 2008 through 2020 from 10 federal agencies. [WNV= West Nile virus; LYME = Lyme disease; ZIKA = Zika virus; POW = Powassan virus].

Vector-borne disease response in the U.S. is a task that requires national policy to fund research and control efforts against both endemic and epidemic diseases. One major opportunity to influence policy authorized to protect against VBD threats is balancing short term versus long term goals (i.e.: fixing versus preventing a problem), and this balancing act can be seen by the trend of federal funding ebbing and flowing with emerging VBDs (Fig. 1). For example, before the emergence of West Nile virus (WNV), no federal funding existed for arbovirus surveillance in the U.S.(Hadler et al. 2015); however, in 1999 after the arbovirus was first detected in New York, congress appropriated annual funding through CDC Epidemiology and Laboratory Capacity (ELC) grants for public health departments to perform surveillance (CDC 2006, Hadler et al. 2015). Additionally, in 2003, the Mosquito Abatement for Safety and Health (MASH) Act was passed into law that authorized ‘grants through the Centers for Disease Control and Prevention for mosquito control programs to prevent mosquito-borne diseases…’ (2003). However, once WNV became endemic, funding for surveillance and control began to wane, and from 2004 to 2012, ELC supported WNV surveillance decreased 61% (Hadler et al. 2014). Additionally, support for the MASH Act subsided, and the legislation ultimately lapsed (Angus King 2019). See Table 1 for a high-level overview of the past and present federal funding avenues discussed in this section.

**Table 1.** Past and present federal funding avenues for vector-borne disease surveillance, management, and research

Legislation	Agency: Sub-agency[and Divisions]	Program
Mosquito Abatement for Safety and Health (MASH) Act Pandemic and All-Hazards Preparedness and Advancing Innovation (PAHPAI) Act Kay Hagan TICK Act in Further Consolidated Appropriations Act	Agency for International Development (USAID) Department of Agriculture (USDA): National Institute of Food and Agriculture; Agricultural Research Service; Animal and Plant Health Inspection Service; Forest Service Department of Defense (DOD): Department of the Navy; Department of the Army; National Geospatial-Intelligence Agency; Department of the Air Force; Uniformed Services University of the Health Sciences Department of Health and Human Services (HHS): Centers for Disease Control and Prevention (CDC) National Center for Emerging and Zoonotic Infectious Diseases (NCEZID) [CDC Division of Vector-Borne Diseases (DVBD); CDC Division of Preparedness and Emerging Infections (DPEI)]; National Institutes of Health Department of State (DOS) Department of The Interior (DOI): Insular Affairs; Nation Park Service; U.S. Fish and Wildlife Service; Departmental Offices; U.S. Geological Survey Environmental Protection Agency (EPA) National Aeronautics and Space Administration (NASA) National Science Foundation (NSF)	Vector-Borne Disease Regional Centers of Excellence Epidemiology and Laboratory Capacity (ELC) Funding for Vector-Borne Diseases ELC for Prevention and Control of Emerging Infectious Diseases

After WNV became endemic, the U.S. experienced a period of relative reprieve from emerging mosquito-borne disease until 2015 when public health was threatened again during the Zika virus pandemic. In 2016, the Strengthening Mosquito Abatement for Safety and Health (SMASH) Act was introduced as a bill in response to Zika virus and as an update to the MASH Act (2016). However, the stand-alone bill was not passed into law and language from the bill did not get passed for three years until the Pandemic and All-Hazards Preparedness and Advancing Innovation (PAHPAI) Act of 2019, well after the initial threat of Zika virus had diminished. Unfortunately, three months after the PAHPAI Act was passed, the COVID-19 pandemic occurred, diverting funds away from VBD efforts. Ultimately, emergency funding in response to the Zika virus outbreak was obtained through a different route in 2016 (Epstein and Lister 2016), but still after the outbreak had occurred.

Mosquitoes are not the only disease vectors threatening Americans that require legislation to provide funding for research and control efforts. Ticks transmit the causative agents of several diseases in the U.S., and federal funding targeting tick control also lags behind emerging/re-emerging tick-borne disease rather than preventing far more prevalent endemic diseases (Fig. 1). For instance, the Kay Hagan TICK Act was signed into law in 2019 after former Senator Kay Hagan contracted and died from Powassan virus (POW) disease (ESA 2020), and even though the original bill specifically calls out reducing the burden of Lyme disease, the success of passing the bill ultimately occurred after the legislation was tied to the high-profile impact of a re-emerging tick-borne virus. And while the threat of POW is real and justifies funding, incidence of endemic Lyme disease is consistently four orders of magnitude greater than POW (Fig. 1).

Whereas the story of federal funding in the last 18 yr is reactive to emerging VBDs, in recent years there have been efforts to create a proactive system. The emergency funds acquired during the Zika virus outbreak were used to establish five Regional Centers of Excellence (COEs) in Vector-Borne Diseases with the goal of preventing and responding ‘to emerging vector-borne disease across the United States’ (CDC 2019). Additionally, the Kay Hagan TICK Act was a step in the right direction because of the legislation’s mandate to create a national strategy to combat vector-borne disease, provide continued support to the COEs, and authorize US$20 million in grants to be awarded to health departments, political subdivisions, and Tribes in underserved areas to address vector-borne diseases. This mandate and these centers are critical to preventing future VBD epidemics and responding to endemic VBD threats. However, whereas authorization of these legislations is the first step, sufficient funding must be appropriated to ensure programs and centers can function and be productive. Additionally, funds need to be established to address endemic VBDs impacting hundreds of thousands of Americans right now, not just address future emerging disease threats.

## A Health Policy Tug-of-War: Competing Public Health Threats

VBDs are not the only concern to human health in the U.S. Millions of residents die annually of chronic disease; heart disease and cancer have been the leading causes of death for years (Kochanek et al. 2017, 2020; Murphy et al. 2018; Xu et al. 2020; Ahmad et al. 2021). Funding for health concerns in the U.S. is divided through the aforementioned legislation pathways, and sometimes there may be a new, or pressing, healthcare or emerging pathogen concerns that divert funding from VBDs, such as the current COVID-19 pandemic, the 2013–2016 Ebola pandemic, and the 2009 H1N1 pandemic (Parpia et al. 2016, Shultz et al. 2016, Xue and Zeng 2019). It is impossible to decide what health threat is the most important for Americans, especially when there is no way to measure the value of a human life. Unfortunately, however, the funding to combat these health threats must be fought for and allocated.

At the current moment as this manuscript is being written, COVID-19 has the attention of the entire world, and consequently, the U.S. Congress. This is without contest, as deaths attributed to this disease reached over 602,000 in the U.S. as we write this manuscript in July 2021, and COVID-19 became the third leading cause of death at this time (Ahmad et al. 2021). Different political parties in the U.S. do not always see eye-to-eye on funding decisions; however, despite differing approaches, most do agree that protecting the people of the U.S. from disease is good. This also applies to VBDs, as evidenced by previous VBD-related legislation—indicating that at the moment, VBD legislation and support appear to be bipartisan. As additional public threats arise, this will be an important aspect for the health of the country as a whole and future VBD legislation.

## Creative and Proactive Approaches to VBD Threats in the U.S.

What successful measures have been taken to be proactive instead of reactive for emerging and re-emerging VBD threats in the U.S.? How can the reactive boom and bust cycle of funding be broken?

### Collaborative Regional and National Centers for Vector-Borne Disease Research

With designated funding, programs can significantly improve efforts to protect the U.S. from VBD threats. In response to the 2016 Zika outbreak and in an effort to strengthen the nation’s capacity to prevent and rapidly respond to such VBD threats, some of the emergency funding obtained by the Obama administration went to support the five Centers for Disease Control and Prevention (CDC) Vector-Borne Disease Regional Centers of Excellence (COEs) in 2017 through 2021 (CDC 2019). The COEs represent close collaborations between mosquito and vector control agencies, academic partners, and public health practitioners at state and local levels, and also provide a funding mechanism for research that is disallowed through the ELC grants. These COEs work to train public health entomologists, vector biologists, and medical providers in VBD-related skills and knowledge; develop and validate effective prediction, prevention, and control methods and tools; and strengthen and expand our communities of practice in VDBs (CDC 2019) Success from the COEs stems from their ability to cross-support stakeholders and pool resources, allowing for effective surveillance and response in areas that otherwise may not have the capacity to act alone or within a timely manner. Regarding training public health entomologists and young professionals in VBDs, each COE has multiple training programs, fellowships, workshops, webinars, boot camps, and even advanced degree options specifically created for this goal. Many of these training opportunities are in-person, but plenty are available online to meet the needs of the new normal during a pandemic. All of the COEs stress that these training opportunities are intended to prepare the next generation of public health entomologists, practitioners, or biologists in the field in detecting and responding to the next VBD threats. Specifically, the internship programs offered through collaborations at the COEs train a new generation of public health entomologists while simultaneously supporting goals of host sites (departments of health, universities, or mosquito and vector control agencies) by increasing their seasonal workforce (MCEVBD 2021, UTMB 2021). Dedicated funding and training grants for students similarly work to ensure the public health entomology workforce remains strong. Fellowships, scholarships, and free trainings seek to lower the barriers of access to technical training, encourage underfunded programs to build capacity and stay up-to-date on nationally guided best practices. Recent examples of these include hands-on trainings to teach tick surveillance and control techniques, insecticide resistance monitoring, and best practices in communication; all tailored to fill the training gaps of the regions served and often provide continuing education units (CEUs) for attendees to further refresh the available workforce (SECVBD 2020, NEVBD 2021a, UTMB 2021). Further, online materials have allowed participants throughout the U.S. and beyond to access high-quality trainings for free or at low cost, while also minimizing time or cost burdens related to travel (PACVEC 2020, MCEVBD 2021, NEVBD 2021a, SECVBD 2021). These resources serve to support local capacity, with a particular effect in supporting partners who may otherwise not receive funding, technical support, or quality training. These opportunities minimize the local effects of otherwise fluctuating or cyclical funding.

Additionally, each initially established COE was charged with a goal to expand, improve, and validate VBD prediction, prevention, and control measures. This goal works hand-in-hand with the third of strengthening communities of practice in VBDs: as methods for prediction and control are improved on, partners will be able to execute effective measures in a timely manner (reducing risk of VBD transmission in residents and constituents) while also increasing communication across academic, governmental, and public health professional institutions. Examples and highlights of these efforts include: (1) the Southeastern Center of Excellence in Vector-Borne Disease’s (SECVBD) mosquito pool testing during the 2020 West Nile and dengue virus outbreaks in southern Florida, ensuring timely turnaround to enable local mosquito and vector control teams to better tailor their efforts (J.C. Beier et al. 2020, unpublished data). (2) The Northeast Regional Center for Excellence in Vector-Borne Disease (NEVBD) provides a pesticide resistance monitoring service as well as screening kits, (NEVBD 2021b) and NEVBD has published manuscripts on validating control measures for other agencies to utilize freely (Burtis et al. 2021, McMillan et al. 2021). (3) The Western Gulf Center of Excellence’s (WGVBD) multiple projects on high-throughput arbovirus detection and diagnostics have been validated through their partnerships with two local public health departments (UTMB 2021), adding rigor and reproducibility to their publications on control methods and insecticide monitoring to enhance control methods (Lee et al. 2020, Juarez et al. 2021). (4) The Midwest Center of Excellence’s (MCEVBD) centralized, public-facing Vector Records Repository which is an open access database on vector records for the entire region, which ensures smooth data sharing and logistics for all researchers and public health professionals in the Midwest (MCEVBD 2021). MCEVBD has also published novel control and risk prediction modeling (Karki et al. 2020, Bron et al. 2021). (5) the Pacific Southwest Center of Excellence in Vector-Borne Disease’s (PACVEC) Border Tick and Rickettsia Surveillance program offers surveillance, free shipping, and free testing of hard ticks in California and Arizona, essentially allowing fluid and standardized tick surveillance in areas where surveillance otherwise may not have been accomplished (PACVEC 2020). Similarly, to their Midwest affiliate, PACVEC has also published novel control and risk prediction modeling (Barker 2019, Holcomb et al. 2021). Lastly, these COEs do no work in silos, and often collaborate to produce high-impact applied entomology articles (Fernandez et al. 2019, Keyel et al. 2021). As the COEs share best practices and protocols through their integrated research, informal peer mentoring, and formal trainings, this allows for greater exchange of ideas while standardizing practices and data throughout each region.

The COEs support efforts in advocacy, education, and outreach, as the network of experts and trainees in VBDs naturally lends itself to share experiences, challenges, and successes of regional concern. Representatives from the COEs have conducted formal educational visits to the Hill to inform national policymakers on VBD issues relevant to their constituencies and to showcase local return-of-investment from these partnerships. The regional COEs, along with the Entomological Society of America (ESA) and other public health groups, also joined forces as the Vector-Borne Disease Network, which makes a concerted effort to inform policymakers (ESA 2021c). Visits such as these have raised awareness for various policies, including the SMASH and Kay Hagan TICK Acts, contributing to the successful passing of these bills. Further, the COEs are dedicated to educating the general public about VBD-related issues, ensuring the dissemination of knowledge from academia to communities served by the COEs. Beyond locally-based outreach, the COEs also worked together with CDC to host webinar series in the spring of 2020 and 2021, which have reached over 2,000 participants, including those at state public health departments, universities, federal agencies, congressional offices, and general citizens (K. Wargo et al. 2020, unpublished data). This indicates a need to ensure current threats and best practices in VBD prevention and control are understood across the societal spectrum, and efforts to increase outreach are at the heart of improving public health from a policy level.

### Mosquito and Vector Control Outreach

All vector control programs, organizations, and abatement districts can benefit from hosting some type of community outreach whether through boots on the ground community engagement, a social media presence, or even education programs for schools. Securing successful community engagement and support for mosquito and vector control programs not only establishes trust but also can help to provide political support (leading to financial resources), citizen scientists for interventions or vector collections, and further dissemination of information regarding the program (Healy et al. 2014, Bartumeus et al. 2019, Rampold et al. 2020). Social media engagement and local media presence are two types of one-way communication processes that can help establish this positive relationship with the community. Examples include local news stories and features, open houses, presence at health fairs, and various social media accounts such as Twitter and Facebook. Although there is little published research on these types of communication, these are recommended initial steps to engage the community. Social media posts have been seen to instill personal responsibility to viewers, which can help in the dissemination of educational information (McLeod-Morin et al. 2020). Further, communication to the public has been deemed successful when messaging is made personal and provides a level of accountability to the general public (Rampold et al. 2020). A successful example of this type of engagement are toolkits and educational curricula developed for elementary, middle, and high school aged children and adolescents. Multiple mosquito and vector control organizations and public health departments have developed these types of programs, successfully integrating applied science and community development. This also leads to an opportunity for increased STEM participation in these younger students. Districts or programs that do not have the resources to hire staff strictly for education can also take advantage of free lesson plans and curriculums available online. These lesson plans were developed to meet curriculum needs regarding science education and were approved by teachers and local schools (Parker et al. 2020a).

Additionally, reaching a younger audience, namely children and adolescents in primary through high school, is highly successful for vector control programs. One mosquito control educational program saw mosquito control and disease knowledge retained for over 5 months post curriculum in primary school-aged children (LaBeaud et al. 2009). A more involved community will lead to continued political and potential financial support for the protection of residents against VBDs.

### AMCA Training and Certification: Surveillance and Control Program

In 2016, as a response to Zika virus emergence, the American Mosquito Control Association (AMCA) was awarded a multi-million dollar contract from CDC to establish training and certificate programs for mosquito surveillance and control (AMCA 2021b). The Zika virus disease outbreak revealed the limited number of medical entomologists and trained individuals in mosquito control available to respond to a large-scale mosquito-borne disease outbreak. The goal of this large AMCA training and certification program was aimed to meet this need for future VBD threats. The program developed includes topics such as insecticide resistance, basic mosquito ecology and behavior, mosquito surveillance, and species specific control following guidelines outlined by AMCA’s Integrated Mosquito Management Curriculum and AMCA’s *Best Practices for Integrated Mosquito Management* manual (AMCA 2021b). Additionally, AMCA launched a virtual E-module training program and train-the-trainer program to increase workforce capacity. The E-Modules emphasize *Aedes* species surveillance and control to help build vector control work force capacity by educating more individuals on managing these vectors, and the train-the-trainer series creates further local capacity by empowering individuals to continue the training within their own organizations. By 2019, these E-Modules had over 1,000 engagements in 43 states and U.S. territories (Walton 2019). The ‘Train the Trainer’ Certification workshops were live events offered at various regional training hubs; attendees were required to complete E-Module 1 before participating. Fourteen ‘Train the Trainer’ workshops were conducted in 10 states from 2017–2018. Proctors of the workshops received Master Training Certifications from the AMCA and CDC. These workshops used the AMCA Best Practices for Integrated Mosquito Management Manual and included a curriculum on surveillance, action thresholds, control, and mapping along with real data and action plans. These workshops resulted in over 400 certifications in 31 states (Walton 2019). The third track has not been formally announced, however, AMCA’s website states that this portion of the program will aid stakeholders with strategic planning and organizational development to help organizations meet long-term goals and needs (AMCA 2021b). This large national-scale training and certification program backed by the CDC most certainly has increased vector control capacity and preparedness in local control programs; this can be investigated in future surveys.

### Developing Easy-to-Deploy Vector Control Tools: the CDC Bottle Bioassay Kits Example

To aid mosquito and vector control organizations in monitoring for insecticide resistance, the CDC developed a rapid and economical assay to assess resistance in any species of insect, including mosquitoes, referred to as the CDC bottle bioassay (CDC 2021c). Organizations within the continental U.S. and its territories are able to request CDC bottle bioassay kits for free which include bottles, insecticides, and a manual for use and safety from CDC’s bottle bioassay webpage (https://www.cdc.gov/mosquitoes/mosquito-control/professionals/cdc-bottle-bioassay.html). Since their development in 1998, bottle bioassays have been refined and streamlined for efficiency (Brogdon and McAllister 1998). Kits are flexible, as these can be applied to any species of mosquito in the continental U.S. reared from a laboratory colony or from the field (McAllister et al. 2020). Organizations that request kits can thus test their local mosquito populations for resistance to gain an understanding of developing resistance patterns and potentially stop continued resistance development using a standardized method that is used widely throughout the U.S. Since the kit program’s inception, 230 CDC bottle bioassays have been sent to different mosquito and vector control organizations or programs, and 192 of those programs (83.5%) are willing to share insecticide resistance results with CDC (J. McAllister 2021, personal communication). Widespread resistance monitoring is highly encouraged, especially for both *Aedes albopictus* (Skuse, 1895) (Diptera: Culicidae) and *Aedes aegypti* (Linneaus, 1762) (Diptera: Culicidae) mosquitoes (CDC 2016). With easy-to-use testing kits, mosquito and vector control agencies can relatively effortlessly begin aiding in this national endeavor.

### Advocacy Programs to Help Break Cyclical VBD Funding

Protecting U.S. citizens requires consistent, sustained funding to perform surveillance, execute interventions, and conduct research to develop innovative tools and control strategies. Advocating and making regulators aware about the issues most important to their voting constituency may help break the reactive cycle of VBD funding. The simplest way to advocate is by reaching out directly to a person’s senate and representative staff and informing them about the issues most important to the advocate. However, programs and partnerships exist that provide advocacy training and support as well. Currently, ESA has a two-year science policy fellowship that recruits entomologists at different career stages, teaches them the tools for successful advocacy, and brings them to Capitol Hill to meet with congressional staffers (ESA 2021a). Additionally, partnering with associations provides further avenues to advocate. The AMCA holds an annual Washington Conference where they take vector control professionals to Washington D.C. and gives them the opportunity to inform staffers about the threat of VBD and how sustained, consistent funding would help protect Americans from the next outbreak (AMCA 2021a). Similarly, the National Pest Management Association holds their annual Legislative Day providing pest control professionals all over the country the opportunity to meet with Senate and Representative staff to discuss the issues most important to them (Harbison 2021). Finally, many of these associations also offer free resources such as infographics, position statements, one-pagers, and more to help individuals advocate most effectively (AMCA 2021a, ESA 2021b).

## Multi-Tiered Focus on Current Vector Control Needs and Infrastructure Capacities: National, Regional, State, and Local Levels

Mosquito and vector control capacities are subject to funding and population-specific perceived need, which are highly cyclical and geographically based (Roehrig 2013). Thus, routine needs assessments can be useful tools for identifying contemporary vulnerabilities, areas for improvement, and prioritizing operational activities. To highlight the variance between multi-tiered infrastructure, we present a side-by-side comparison of three contemporary needs assessments (Table 2). As noted in the table and related publications, one critical challenge is the mismatched framework of vector control organizations. Mosquito control organizations are funded through a variety of sources: health departments, city planning, police departments, public works departments, independent mosquito control districts, state funded abatement districts, and other avenues. This decentralization creates considerable challenges for disseminating emergency funding, continuing technical education, and community resource support. AMCA, along with regional and state mosquito control associations, attempts to provide a connecting backbone infrastructure; however, these non-profit organizations do not exist specifically for ticks or other important vectors.

**Table 2.** Capabilities in mosquito and vector control vary widely in the United States, with most agencies performing routine mosquito surveillance and chemical abatement; however few programs are capable of performing pathogen testing and even fewer can test for insecticide resistance

	National Survey (NACCHO 2017)	Regional Survey^*a*^ (Johnson et al. 2020)	State Survey^*a*^ (Moise et al. 2020)
Response rate	57% (1,083/1906)	45% (150/333)	49% (44/90)
Respondents organizational type	• Mosquito control districts • Local health departments • Other city/local government agencies	• Local employees registered with state vector control agency list-servs • Local health departments • Other city/local government agencies	• Municipality • County department • Operates under Board of County Commissioners • Independent special taxing district
Percent of respondents a part of local health department	53%	47%	9%
Performs routine mosquito surveillance	54%	70%	82%
Performs chemical abatement (larvicide and/or adulticide application)	68%	84%	97%
Performs pesticide resistance testing in-house	14%	56%	Not assessed
Performs mosquito pathogen testing	Not assessed	30%	49%

^*a*^Regional survey is based on the Southeastern U.S. and the state survey is based in Florida.

A second capacity challenge is the inability to systematically detect and respond to vectors. The cyclical nature of vector control response inherently creates opportunities to breed and expand resistant populations and invasive species without consistent and monitored surveillance and control efforts. For example, insecticide resistance monitoring is not performed consistently or widely in the U.S. (NACCHO 2017). As evidenced in Table 2, approximately half of southeastern vector control agencies monitor for resistance in mosquitoes, in sharp contrast to 14% nationally. We know that vectors, including mosquitoes, do not respect regional boundaries, and this inconsistency could pose a threat to resistance management in other parts of the nation.

A third challenge is the interpretation of vector control needs assessments. For example, one might interpret the finding that ‘half of southeastern agencies perform insecticide resistance monitoring compared to 14% nationally’ as the concern for insecticide resistance is greatest in the northern, central, or western regions, due to their lower insecticide resistance testing. Conversely, one might interpret the statement as the concern for insecticide resistance is greatest in the southeastern region and thus testing had been made a priority effort in that region. Creating state, regional, or even national interdisciplinary scientific advisory boards for the interpretation of routine needs assessments, and making routine needs assessments a priority activity, can begin to address and overcome these challenges and create opportunities for realistic solution implementation of timely needs.

On a positive note, a majority of vector control agencies responded in all three needs assessments that they do perform routine mosquito vector surveillance, a key function for outbreak preparedness (Table 1). Tick surveillance was only included in the Johnson et al. (2020) needs assessment (Johnson et al. 2020), and this surveillance activity was sparse compared to mosquito surveillance. The lack of tick surveillance is a critical issue, as tick-borne diseases represent 93% of the 70,567 cases of nationally notifiable vector-borne diseases reported to CDC in 2019 (CDC 2021b). Additionally, the regional and state needs assessments revealed 30–49% of vector control agencies performed mosquito pathogen testing, whereas the national survey did not assess testing. This component of surveillance is also crucial for evidence-based mosquito control as simply the presence of a vector does not infer pathogen presence.

## Future Directions and Concerns

On a national level, the U.S. struggles to meet the basic vector control competencies of performing routine mosquito surveillance to guide abatement application, the ability to perform insecticide resistance to inform abatement selection, and even the capacity to apply chemical control (NACCHO 2017). When considering control of other important vectors, such as ticks, triatomines, fleas, etc., the U.S. performs even worse on these metrics (Petersen et al. 2019). In fact, tick-borne human disease cases have steadily doubled in the past decade (Rosenberg et al. 2018), and the recent introduction of the first documented invasive tick species in 80 yr (Beard et al. 2018) highlights the need to expand surveillance and abatement capacities beyond domestic mosquitoes. As only 1 of the 17 nationally reportable vector-borne diseases has a widely available vaccine and 7 of the 17 pathogens have available therapeutics (2021), the emphasis on vector control and prevention is critical. Even with the knowledge of needed structural improvements related to VBD preparedness and surveillance, the simple lack of communication and cooperation among U.S. federal agencies regarding VBD capacity and needs is staggering. Unfortunately, a lack of unified support within the federal government has led to confusing and difficult to manage regulatory processes and a heavily politicized litigation-driven pesticide registration process. But this lack of communication does not only exist on the federal level: even in the state and local government levels fragmentation and jurisdiction issues exist that can result in lack of communication or detract from the common goal of VBD prevention and control.

The CDC published the first national guidelines for vector-borne disease prevention and control in 2020 to help initiate communication and collaboration throughout all levels of government (2020). This framework lists five principal goals centered around increased epidemiologic and transmission knowledge, improved vector control and technical education, enhanced diagnostic tools, generation of novel therapeutics, and public education dissemination. Additionally, various challenges are discussed about the U.S.’s ability to detect and respond to vector-borne disease threats, which if addressed will significantly improve the ability to protect against the next VBD outbreak (CDC 2020c). Some of these challenges include the nation’s stressed surveillance systems, limited capacity to respond to outbreaks, and the lack of interconnected, quality data. The aforementioned measures, like the COEs, are steps in the right direction to addressing these challenges.

Additional challenges include disparities related to vector-borne disease capacity, the general lack of diversity in the medical entomology or vector control field, and insecticide resistance development. Although routine mosquito surveillance and pathogen testing are somewhat common in some vector control agencies (Table 2), both of these can be expensive processes due to salary, equipment, and chemical reagent costs, potentially creating a disparity in vector control program efficacy dependent on federal funding if locally-appropriated funds are not sufficient. Some opportunities for overcoming this financial disparity include additional funding mechanisms such as the National Science Foundation’s Established Program to Stimulate Competitive Research (NSF EPSCoR), the National Institute of Health’s R-15 Research Enhancement Award, or the 2019 Fostering Undergraduate Talent by Unlocking Resources for Education (FUTURE) Act, all of which aim to strengthen research programs at smaller institutions or programs that do not typically receive large grants and expose students and young researchers to rigorous, applied scientific research.

The aforementioned booms and busts in federal funding lead to unstable employment year to year, or at least uncertain longevity in the field. The majority of highly skilled STEM graduates—across all genders and races/ethnicities—never work in highly skilled STEM jobs and are just as likely to be unemployed as non-STEM graduates (Smith and White 2019). With a job market that is based on an ephemeral boom and impending bust, STEM desirability may not be high in the current climate (Smith and White 2019). However, the severe imbalance of minorities and women in vector control-related disciplines yields a disparate environment, one that continues the same cycle of imbalance and limits the potential of the field itself because diversity leads to diversity of thought and diversity of thought leads to innovation (Xu 2015, Orfinger 2020). In general, minorities report fewer same-race role models, have fewer exposures to, and less comfort in ecology and evolutionary biology fields compared to non-Hispanic white Americans (Thébaud and Charles 2018, O’Brien et al. 2020). In the entomology and parasitology fields specifically, the NSF found that only 2.3% of graduate students reported being of Black race and 4.9% of Hispanic/Latinx ethnicity in 2016 (NSF 2019, Orfinger 2020). Addressing this unequal access to higher education requires acknowledgment of and efforts to combat the driving forces of cultural, structural, and institutional barriers within the educational system, which is beyond the scope of this paper (Abramson et al. 2013, López-Uribe 2020, Orfinger 2020). Similarly, following graduation, females in STEM disciplines are less likely to pursue advanced positions or remain in these careers for as long as their men counterparts (Xu 2015, Thébaud and Charles 2018, Jasko et al. 2020). Additionally, female entomologists, despite representing approximately 50% of all doctoral graduates, are significantly underemployed in entomology disciplines including government and academia compared to men (Walker 2018).

Programs that support mentor and networking opportunities for underrepresented groups could help aid in encouraging minorities in STEM to begin pursuing these careers earlier (Jasko et al. 2020). At all educational levels below college, it is widely believed that fostering girls’ and minority interest in STEM at a younger age will only increase the proportion of motivated women and minorities in the field (Xu 2015). The CDC’s national framework for vector control could lead to resources being dedicated to include school-aged education programs directly aimed at fueling the medical entomology pipeline regarding minorities. At the college level, efforts such as career offices at institutions of higher education that assist female students could increase motivation for technical knowledge and skills, reduce uncertainty about the possibilities in the job market for women, and increase support for women managing both careers and homes (Xu 2015, Thébaud and Charles 2018, Jasko et al. 2020). In the workplace, cultural support such as ESA’s annual conference event ‘Women in Entomology Breakfast’ serves as a model for potential expansion in the entomological field. Both AMCA and ESA also have education days geared towards allowing students the ability to meet experts in the medical and general entomology fields. Similar programs for minority students to meet experts in STEM fields may aid in filling the gap in STEM representation. Efforts should be made throughout the current vector control environment that encourages the participation of all ethnic groups and sexual identities to cultivate an environment of diversity and inclusion, ultimately benefiting the future of the field (Smith and White 2019).

Lastly, insecticide resistance is a significant concern for the future of vector control efforts nationally and globally. Insecticide resistance is the ‘ability in a strain of insects to tolerate doses of toxicants which would prove lethal to the majority of individuals in a normal population of the same species’ (WHO 1957). In a naïve population of insects—insects never exposed to the insecticide-susceptibility is normally distributed, with equal proportions of the population dying at very low and surviving at very high concentrations of insecticide. During control efforts, those few individuals in the population that survive go on to be the progenitors of the new population, passing on those heritable traits that allowed them to survive. With time, the new population can survive exposure to much higher concentrations of the insecticide than the previous generations and can result in control failures. The underlying mechanisms of insecticide resistance fall within four general categories: reduced target site sensitivity, decreased cuticular penetration, altered enzymatic activity, and/or altered behavior (Martinez-Torres et al. 1999, Wood et al. 2010, Gordon and Ottea 2012, Gatton et al. 2013). Additionally, resistant populations can exhibit two or more of these mechanisms simultaneously (Awolola et al. 2009), and life history tradeoffs between fitness costs and mechanisms of resistance exist (Brito et al. 2013).

Insecticide resistance management (IRM), ‘is the scientific approach to managing pests over the long run so that resistance does not interfere with our ability to accomplish our goals’ (Onstad 2008). To develop successful IRM strategies, understanding the mode of action of the insecticide, mechanisms of resistance, and life history tradeoffs are important to create strategies that will prolong the usefulness of current control tactics and prevent control failures. Given the relative paucity of new active ingredients available (Bunge and McKay 2017) and the incredibly high price tag to bring a new product/active to market (Whitford et al. 2006) preserving the usefulness of our currently available actives must be a high priority for all mosquito abatement programs. Thus, sustained federal funding must be provided to equip mosquito and vector control programs with the capabilities necessary to quantify levels and characterize mechanisms of resistance to respond to insecticide resistance threats in real-time before an insecticide active ingredient can no longer control a population of mosquitoes. Without proactive, effective tools, mosquito abatement programs cannot protect the public’s health from VBD. The free CDC bottle bioassay kits will be moot if federal funding is not appropriated and the insecticide industry is not supportive of mosquito and vector control agencies actually combating resistance development.

As highlighted in the CDC’s national framework for vector control, most of these concerns are not just a problem for the U.S. (CDC 2020c); however, the mechanisms for enacting change and dedicating funding for vector control and research efforts vary widely across the globe. As multiple federal departments are called to action, international efforts must also be put forth to combat these issues, including communication between ministries or departments of health, agriculture, and environmental health. Infrastructure and political instability can hinder these international efforts, especially when vectors and undiagnosed infirmed persons are able to traverse international borders easily through travel and trade (Cherry et al. 2018, Boggild et al. 2019, Maljkovic Berry et al. 2020).

Pathogens themselves are not the only threats to public health, as we have seen the movement of invasive vector species across international borders which significantly impact agriculture and health in the U.S. The following examples exemplify this concern. (1) *Haemaphysalis longicornis* Neumann, 1901 (Ixodida: Ixodidae) the Asian longhorned tick (introduced in the 2010s) which has spread to 15 states and has the potential to hurt the livestock industry (Beard et al. 2018, USDA 2021). (2) The re-established population of New World screwworm, *Cochliomyia hominivorax* (Coquerel, 1858) (Diptera: Calliphoridae), in southern Florida in 2016 which put local wildlife, livestock, pets, and humans at significant risk for infestation—this invasion was addressed and eliminated (Hennessey et al. 2019, Parker et al. 2020b). (3) Lastly, *Ae. albopictus* (introduced in 1986 in Texas) which has vectored multiple arboviruses in the U.S. and impacted the vector distribution within its associated microhabitat (Sprenger and Wuithiranyagool 1986, Lounibos et al. 2001, Braks et al. 2004).

There will be additional vector-borne disease threats in the near future, whether in the form of the vectors themselves or pathogens crossing borders. Climate change, urbanization, and international travel create favorable conditions for populations of vectors to grow and increase the chance that a person will come into contact with a possible vector. The past 20 yr of VBD policy and funding demonstrate that VBDs will not dissipate, and the only way to solve the future directions and concerns outlined above is through stable, consistent funding. As a nation, we need to take the steps necessary to ensure that we are prepared to combat these public health threats by providing sufficient, preventative funding.

## Funding

This research was supported in part by the United States Centers for Disease Control and Prevention (CDC) Grant 1U01CK000510: Southeastern Regional Center of Excellence in Vector Borne Diseases: The Gateway Program. The CDC did not have a role in the design of the study, the collection, analysis, or interpretation of data, nor in writing the manuscript.
